# Addressing overfitting bias due to sample overlap in polygenic risk scoring

**DOI:** 10.1002/alz.70109

**Published:** 2025-04-06

**Authors:** Seokho Jeong, Manu Shivakumar, Sang‐Hyuk Jung, Hong‐Hee Won, Kwangsik Nho, Heng Huang, Christos Davatzikos, Andrew J. Saykin, Paul M. Thompson, Li Shen, Young Jin Kim, Bong‐Jo Kim, Seunggeun Lee, Dokyoon Kim

**Affiliations:** ^1^ Graduate School of Data Science Seoul National University Seoul Republic of Korea; ^2^ Department of Biostatistics, Epidemiology and Informatics, Perelman School of Medicine University of Pennsylvania Philadelphia Pennsylvania USA; ^3^ Department of Medical Informatics Kangwon National University, College of Medicine Chuncheon Republic of Korea; ^4^ Samsung Advanced Institute for Health Sciences and Technology (SAIHST) Samsung Medical Center Sungkyunkwan University Seoul Republic of Korea; ^5^ Indiana Alzheimer's Disease Research Center Indiana University School of Medicine Indianapolis Indiana USA; ^6^ Center for Computational Biology and Bioinformatics Indiana University School of Medicine Indianapolis Indiana USA; ^7^ Department of Electrical and Computer Engineering University of Pittsburgh Pittsburgh Pennsylvania USA; ^8^ Center for Biomedical Image Computing and Analytics Perelman School of Medicine University of Pennsylvania Philadelphia Pennsylvania USA; ^9^ Department of Radiology and Imaging Sciences and Indiana Alzheimer Disease Center Indiana University School of Medicine Indianapolis Indiana USA; ^10^ Department of Medical and Molecular Genetics Indiana University School of Medicine Indianapolis Indiana USA; ^11^ Imaging Genetics Center Laboratory of Neuro Imaging Department of Neurology & Psychiatry UCLA School of Medicine Los Angeles California USA; ^12^ Division of Genome Science Department of Precision Medicine National Institute of Health Cheongju Republic of Korea; ^13^ Institute for Biomedical Informatics Perelman School of Medicine University of Pennsylvania Philadelphia Pennsylvania USA

**Keywords:** Alzheimer's disease, genetic risk factor, polygenic risk scores, precision medicine, sample overlap

## Abstract

**INTRODUCTION:**

Numerous studies on Alzheimer's disease polygenic risk scores (PRSs) overlook sample overlap between International Genomics of Alzheimer's Project (IGAP) and target datasets like Alzheimer's Disease Neuroimaging Initiative (ADNI).

**METHODS:**

To address this, we developed overlap‐adjusted PRS (OA PRS) and tested it on simulated data to assess biases from different scenarios by varying training, testing, and overlap proportions. OA PRS was used to adjust for sample bias in simulations; then, we applied OA PRS to IGAP and ADNI datasets and validated through visual diagnosis.

**RESULTS:**

OA PRS effectively adjusted for sample overlap in all simulation scenarios, as well as for IGAP and ADNI. The original IGAP PRS showed an inflated area under the receiver operating characteristic (AUROC: 0.915) on overlapping samples. OA PRS reduced the AUROC to 0.726, closely aligning with the AUROC of non‐overlapping samples (0.712). Further, visual diagnostics confirmed the effectiveness of our adjustments.

**DISCUSSION:**

With OA PRS, we were able to adjust the IGAP summary‐based PRS for the overlapped ADNI samples, allowing the dataset to be fully used without the risk of overfitting.

**Highlights:**

- Sample overlap between large Alzheimer's disease (AD) cohorts poses overfitting bias when using AD polygenic risk scores (PRSs).
- This study highlighted the effectiveness of overlap‐adjusted PRS (OA ‐PRS) in mitigating overfitting and improving the accuracy of PRS estimations.
- New PRSs based on adjusted effect sizes showed increased power in association with clinical features.

## 1 BACKGROUND

For complex disease research, the polygenic risk score (PRS) has emerged as a promising early detection and prevention method. As the performance of PRS improves with larger training datasets, researchers often use comprehensive genetic consortia summary statistics to construct PRSs.^1^ These consortia typically encompass a broad spectrum of existing genome‐wide association studies (GWASs), which means the GWAS summary data may include samples from the target dataset intended for evaluating the PRS's performance and utility.^2^, ^3^ This is particularly common in Alzheimer's disease (AD) research, in which summary statistics from the International Genomics of Alzheimer's Project (IGAP) are mainly used as a training dataset. IGAP compiles data from various consortia, including the Alzheimer's Disease Genetic Consortium; the Cohorts for Heart and Aging Research in Genomic Epidemiology Consortium; the European Alzheimer's Disease Initiative; the Genetic and Environmental Risk in Alzheimer's Disease (GERAD); the Alzheimer's Disease Neuroimaging Initiative (ADNI); and the Religious Orders Study/Memory and Aging Project (ROSMAP). In many PRS studies in which IGAP summary statistics are used as a training dataset, one of these cohort datasets is chosen as the target dataset for the evaluation. The overlapping of data presents a widespread challenge across different research consortia, such as IGAP^4^ and ADNI,^5^, ^6^ IGAP and ROSMAP,^6^, ^7^ the UK Biobank (UKBB) and the Psychiatric Genomics Consortium (PGC),^8^ the Bulgarian schizophrenia cohort and PGC,^9^ the Danish high‐risk and resilience study and PGC,^10^ GWAS & Sequencing Consortium of Alcohol and Nicotine and UKBB,^11^ and the Asian Genetic Epidemiology Network Type 2 Diabetes (AGEN‐T2D) consortium and the Korean Genome and Epidemiology Study (KoGES).^7^, ^8^, ^9^, ^10^, ^12^, ^13^, ^14^, ^15^, ^16^

Many studies have used ADNI as the target dataset, despite encountering ≈ 300 to 400 sample overlaps with training data, varying by the data source. Researchers who considered using the IGAP summary and ADNI cohort together had difficulties in removing overlapped samples due to consent issues and the complexity of institutional restrictions. Some of them have acknowledged this overlap but considered its impact negligible,^17^ whereas others have attempted to address it by excluding the overlapping samples from the ADNI target cohort.^18^ However, removing samples from the target dataset is suboptimal as it diminishes the statistical power for downstream analysis, particularly when linking genetic components with imaging, clinical, and biospecimen data such as those in ADNI. This issue is also observed in the case of other datasets like ROSMAP^19^ and the Wisconsin Alzheimer's Disease Research Center (WADRC).^20^ On the other hand, using the whole target dataset, including overlapping samples, leads to inflated results in PRSs. This issue is highlighted in recent discussions, showing that PRS results using IGAP summary statistics are prone to inflation,^6^ though no specific solution has been proposed.

Although the risk of overfitting due to sample overlap is acknowledged, there is a call for more comprehensive investigations to find effective ways to adjust for this bias.^6^ For instance, a simulation experiment in this study in which 300 cases and 300 controls from the target data were included in the training dataset of 38,000 samples, average inflation in area under the receiver operating characteristic (AUROC) was as large as 10.59%. Ideally, removing overlapping individuals from the association study would correct this; however, such an approach is only possible with access to individual‐level data when the target does not completely overlap with training data or summary statistics generated from within‐cohort summary statistics. Because IGAP's individual‐level genetic data are not publicly accessible, it is not possible to regenerate the summary statistics by excluding specific overlapping samples.

To address this systematic bias, we propose a sample overlap adjustment procedure, overlap‐adjusted (OA) PRS. This approach aims to adjust sample overlap at the level of GWAS summary statistics and includes visual diagnostics to help validate the adjustments made. Our study extensively investigates how OA PRS can mitigate the effects of sample overlap in the construction of practical PRS. We used simulation results based on the UKBB coronary artery disease (CAD) phenotype and the AGEN‐T2D consortium to quantify the sample overlap bias and to verify OA PRS adjusts for sample overlap. Then, we applied OA PRS to IGAP to adjust for sample overlap with ADNI and verified it using visual diagnostics.

## 2 METHODS

### 2.1 OA PRS

**FIGURE 1 alz70109-fig-0001:**
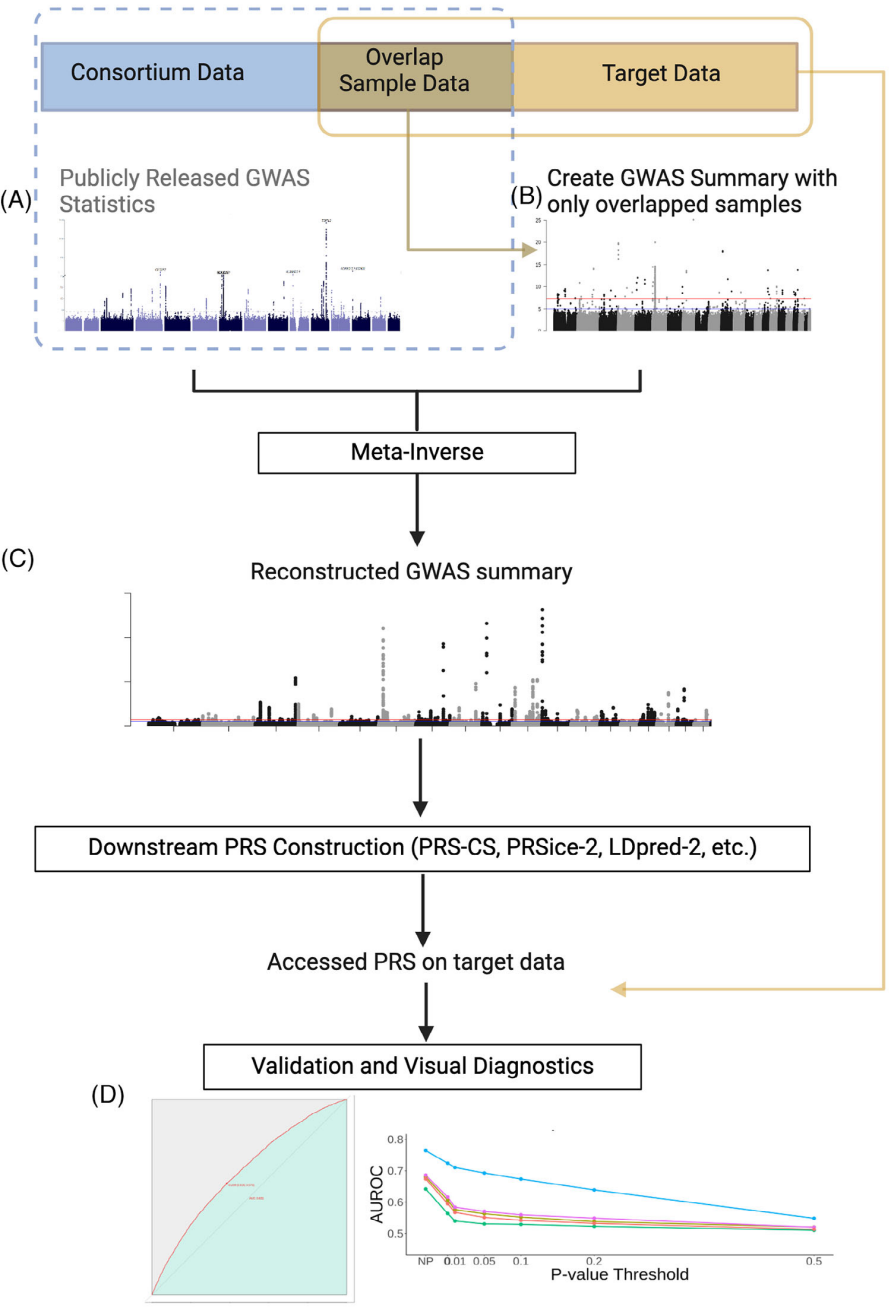
Overview of OA PRS. A, B, Summary information preparation step with publicly released GWAS and overlapped sample summary C, Reconstruction of GWAS summary with OA‐PRS. D, Validation and visual diagnostics of adjusted PRS. AUROC, area under the receiver operating characteristic; GWAS, genome‐wide association study; OA PRS, overlap‐adjusted polygenic risk score.

The main purpose of the OA PRS procedure is to reconstruct GWAS summary data from the consortium summary using overlap sample data from the target dataset. This reconstructed summary is designed to resemble the GWAS summary generated by removing overlapping samples from the consortium data. As shown in Figure 1, OA PRS consists of four main steps: summary information preparation, (B) sample overlap adjustment using meta‐inverse, (C) PRS construction, and (D) validation and visual diagnostics. In the summary information preparation step, genetic association levels from overlapped samples are estimated using individual genotypes from target data, joined with publicly released consortium GWAS summary data. Then, OA PRS reconstructs OA effects by the meta‐inverse approach. After the adjustment, we can follow a standard PRS procedure by applying conventional PRS construction methods such as PRS‐CS,^21^ PRSice2,^22^ and LDpred2.^23^ Last, validation of PRS is performed using both overlapped and non‐overlapped target data. Also, OA PRS provides visual diagnostics to assess whether the adjustment adequately reduced the overfitting bias due to sample overlap.

**RESEARCH IN CONTEXT**

1. **Systematic review**: The authors reviewed the literature using PubMed and Google Scholar. Few studies have examined the problem of sample overlaps in polygenic risk score (PRS) construction. The authors found Alzheimer's disease (AD) studies in which the sample overlap was ignored, possibly leading to inflated results. A recent study published also concluded that PRS results in most AD databases using International Genomics of Alzheimer's Project (IGAP) summary statistics are inflated because of sample overlap between IGAP and target datasets used in various studies. The authors also found a recently published tool, Erase Sample Overlap and Relatedness (EraSOR), which also aims to address the same problem.
2. **Interpretation**: Our simulations indicated that there was significant inflation even when the overlap sample size was small, and the inflation increased as the overlap sample size increased. The overlap‐adjusted PRS procedure described in this study was able to adjust for the overlapped samples and control the inflation.
3. **Future directions**: Our work solves the problem when we know which samples in the target dataset overlap with the consortium summary statistics, but this information is not always available. In the future we plan to develop methods to adjust for sample overlap when the samples are unknown.

### 2.2 Meta‐inverse approach

Meta‐inverse methods exclude the genome‐wide association contributions of overlapped samples from a consortium GWAS, leaving only the effects of non‐overlapped samples. This approach reverts meta‐analysis procedures, such as inverse‐variance weighting (IVW) and *Z* score–based methods.^24^, ^25^ If the consortium GWAS were conducted through meta‐analysis and the same summary data of the overlapped samples used in the meta‐analysis were used in the meta‐inverse, this approach would be the exact approach in the sense that the output summary statistics are the same as the meta‐analysis results without the overlapped samples.[Fig alz70109-fig-0001]

In the IVW‐based approach, effect size adjustment is performed by reversing the IVW, which inverts the meta‐analysis procedure. Suppose $b_{\mathrm{all}}^{j},\;\sigma_{\mathrm{all}}^{j},\;p_{\mathrm{all}}^{j},z_{\mathrm{all},}^{j}$ respectively, represent the estimated effect size, standard error, *P* value, and standardized *Z* score of marker *j* in the consortium GWAS, and the corresponding overlapped and adjusted summary statistics are denoted as $b_{o}^{j},\;\sigma_{o}^{j},\;p_{o}^{j},z_{o}^{j}$ and $b_{s}^{j},\;\sigma_{s}^{j},\;p_{s}^{j},z_{s}^{j}$. The IVW meta‐analysis is applied as follows:

$$\begin{array}{ccc} b_{\mathrm{all}}^{j} & = & \frac{b_{o}^{j}/{\left(\sigma_{o}^{j}\right)}^{2}+b_{s}^{j}/{\left(\sigma_{s}^{j}\right)}^{2}}{1/{\left(\sigma_{o}^{j}\right)}^{2}+1/{\left(\sigma_{s}^{j}\right)}^{2}}\\ \frac{1}{{\left(\sigma_{an}^{j}\right)}^{2}} & = & \frac{1}{{\left(\sigma_{o}^{j}\right)}^{2}}+\frac{1}{{\left(\sigma_{s}^{j}\right)}^{2}} \end{array}$$

By inverting this procedure, we obtain

$$\begin{array}{ccc} {\left(\sigma_{s}^{j}\right)}^{2} & = & \frac{1}{1/{\left(\sigma_{\mathrm{all}}^{j}\right)}^{2}-1/{\left(\sigma_{o}^{j}\right)}^{2}}\\ b_{s}^{j} & = & {\left(\sigma_{s}^{j}\right)}^{2}\;\times \left(\frac{b_{\mathrm{all}}^{j}}{{\left(\sigma_{\mathrm{all}}^{j}\right)}^{2}}-\frac{b_{o}^{j}}{{\left(\sigma_{o}^{j}\right)}^{2}}\right) \end{array}$$

The *Z* score method uses standardized scores from each GWAS, so the estimated $z_{s}^{j}$ is expressed as when the sample size was given as $n_{\mathrm{all}},\;n_{o},\;n_{s}$ for each group.

$$\;z_{\mathrm{all}}^{j}=\frac{\sqrt{n_{o}}z_{o}^{j}+\sqrt{n_{s}}z_{s}^{j}}{n_{o}+n_{s}}$$

$$\;z_{s}^{j}=\left(n_{\mathrm{all}}z_{\mathrm{all}}^{j}-\sqrt{n_{o}}z_{o}^{j}\right)\;/\sqrt{n_{s}}$$

For standard error and effect size estimation, we assumed the response variable is scaled to have a mean of 0 and a standard deviation (SD) of 1. Simple simulation results using the meta‐inverse approach are presented in the Method S1 and Figure S1 in supporting information.

### 2.3 De‐correlation approach

De‐correlation transforms target and consortium effect sizes to make the correlation between two summaries become 0 as:^26^

$$\;\left[\begin{array}{c} \zeta_{\mathrm{all}\;}^{j}\\ \zeta_{o}^{j} \end{array}\right]=\left[\begin{array}{cc} 1 & \;\frac{n_{c}}{\sqrt{n_{\mathrm{all}}n_{o}}}\mathrm{cor}\left(Y_{1},Y_{2}\right)\\ \frac{n_{c}}{\sqrt{n_{\mathrm{all}}n_{o}}}\mathrm{cor}\left(Y_{1},Y_{2}\right)\; & 1 \end{array}\right]\;\left[\begin{array}{c} z_{\mathrm{all}}^{j}\\ z_{o}^{j} \end{array}\right]$$

where $n_{c}$ denotes the joint sample size between two GWAS. After the transformation, the new GWAS effect $\zeta_{\mathrm{all}\;}^{j}$ becomes independent of the overlapped effect $\zeta_{o}^{j}$, under the normal assumption, indicating overlapped samples in the target set are adjusted. Erase Sample Overlap and Relatedness (EraSOR) extends this method by estimating the sample overlap proportion via linkage disequilibrium score regression (LDSC). Let the heritability and intercept terms from bivariate and univariate LDSC between the consortium and target be denoted as $\hat{h_{b}^{2}},\hat{h_{\mathrm{all}}^{2}},\hat{h_{o}^{2}},\hat{I_{b}},\hat{I_{\mathrm{all}}},\hat{I_{o}}$. Then, the relationship between the bivariate and univariate LDSC can be expressed as:

$$\;\hat{I_{b}}=\frac{n_{c}}{\sqrt{n_{1}n_{2}}}+\frac{\hat{\rho_{g}}\sqrt{n_{1}n_{2}}}{n_{1}+n_{2}}\sum_{i=\mathrm{all},o}\frac{\hat{I_{i}}-1}{\hat{h_{i}^{2}}}$$

We applied EraSOR as a representative de‐correlation approach for comparison to the meta‐inverse subtraction approach. However, EraSOR is not advised to be used for sample size *n* < 1000.

### 2.4 Visual diagnosis procedure

Sample overlap adjustment methods require verification to check if the overfitting bias is removed because they cannot explicitly remove the overlapped samples from the consortium GWAS. In the OA PRS, we suggest a simple heuristic approach that gradually removes associative variants from the consortium and the target GWAS. The underlying idea is that PRS will have no predictive power after removal if there is no overfitting.

A more detailed process follows: First, remove highly associated variants and their neighbors that can be correlated using independent linkage disequilibrium block information. Second, filter out variants with a medium association level with multiple *P* value thresholds. Finally, the trajectory of prediction measures using filtered variants lets us decide the level of overfitting on adjustment methods. Practitioners can use arbitrary thresholds such as *P* = 0, 0.01, 0.05, 0.1, 0.2, and 0.5 to exclude associated variants.

### 2.5 UKBB CAD simulation analysis

We used imputed genotyping data from UKBB to generate various sample overlap scenarios to test how such overlap affects a PRS. After applying genotype quality control (Methods S1), this analysis included 368,584 unrelated samples and 9,804,297 markers. The phenotype for CAD was defined using a composite of angina and myocardial infarction. Further information on phenotype definition is available in Yun et al.^27^ We split the UKBB CAD data into training and testing set partitions with split ratios of 50:50, 60:40, 70:30, 80:20, and 90:10. The sample size in each split is given in Figure S2 in supporting information. In addition, we generated overlap partitions by randomly selecting 1%, 3%, 5%, 10%, 20%, 40%, 60%, and 90% of samples from the testing dataset and merging them with the training dataset. In total, we generated 40 different training and testing datasets. We then conducted GWAS using SAIGE on the training datasets and overlapped samples selected from the test datasets.^28^ The summary statistics files generated were used as input to OA PRS to remove the bias due to sample overlap.

To create scenarios with a small testing sample size, we randomly sampled from UKBB CAD data to build a training set consisting of 30,000 controls and 8000 cases and a testing set with 500 controls and 500 cases. Five overlapped datasets were generated by randomly sampling controls and cases from the training dataset in increments, starting at 100 samples each, and merging them into the testing dataset. We repeated this process 10 times to create 10 different sets of randomly sampled datasets. The OA PRS procedure was then applied as described above, and AUROC was evaluated in all tested scenarios. PRS was generated using PRS‐CS,^21^ PRSice2,^22^ and LDpred2^23^ (Methods S1).

The PRS association was run using CAD as our binary outcome and PRS scores as predictors on total (overlapped + non‐overlapped) samples. Using the same model, the AUROCs were calculated on total, overlapped, and non‐overlapped samples, as shown in Figure 1A. We also calculated odds ratio (OR) for all simulations (Figure S3 in supporting information).

### 2.6 IGAP and ADNI overlap analysis

**FIGURE 2:**
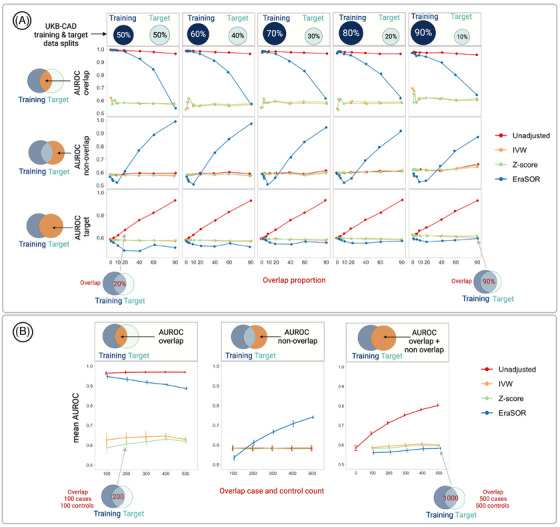
Results from simulation studies using the UKB‐CAD dataset. A, Inflation in AUROC values for overlap, non‐overlap, and target (overlap + non‐overlap) data as the proportion of overlap samples increases and with adjustment by three methods—IVW, *Z* score, and EraSOR. Training and target data splits are shown above the plots (50:50, 60:40, 70:30, 80:20, and 90:10). The *y* axis plots AUROC and the *x* axis the percentage of overlap samples from the target dataset (1%, 3%, 5%, 10%, 20%, 40%, 60%, and 90%). B, Inflation in AUROC values when using a small target dataset. The error bars show 95% confidence intervals for 10 simulation runs. AUROC, area under the receiver operating characteristic; EraSOR, Erase Sample Overlap and Relatedness; IVW, inverse variance weighting; UKB‐CAD, UK Biobank coronary artery disease.

ADNI is a multisite longitudinal study on AD. In that study, the HiSeq2000 platform was used to generate whole‐genome sequencing data for 1566 samples. After quality control (Methods S1), 1545 samples and 15,456,635 variants were retained for the present analysis. We then generated a PRS based on IGAP summary statistics from Kunkle et al.,^15^ which includes 327 ADNI samples, and evaluated it in overlapped and non‐overlapped samples. We ran GWAS using SAIGE separately on the overlapped samples to generate overlap‐only summary statistics, which were used as input to OA PRS to remove bias from the IGAP summary statistics. Subsequently, the PRS association was run using AD as the binary outcome, PRS scores as the predictor, and adjusting for age, sex, and the first four principal components (PCs) as covariates on total (all ADNI samples) samples. Using the same model, the AUROCs were calculated on total samples, overlapped samples (ADNI samples overlapped with IGAP), and non‐overlapped samples (ADNI samples not overlapped with IGAP) as shown in Figure 2A.

### 2.7 AGEN‐T2D and KoGES overlap analysis

In this analysis, the PRS was calculated from the AGEN‐T2D summary statistics of 433,540 participants with 11,825,585 variants, including the KoGES study; these statistics included *Z* score, effect size, standard error, minor allele frequency (MAF), and *P* value. KoGES data provided by the Korean National Institute of Health consists of categorized variables such as genotype array, clinical measurements, and disease status from interviews. The 72,298 individuals in the data come from three cohorts: Ansan/Ansung (AS; *n* = 5493), Rural (*n* = 8105), and Urban (*n* = 58,700). We extracted T2D status as the main phenotype and selected the AS cohort for the experiment on diagnosis, in which three samples were excluded due to missing responses. After quality control (Method S1), the GWAS summary statistics on 8,055,986 variants of the AS cohort were generated with SAIGE and adjusted to the original AGEN‐T2D summary statistics, resulting in 7,731,011 variants. During the diagnosis, highly associated markers were removed (*P* = 1e‐04), and thresholds of *P *= 0, 0.01, 0.05, 0.1, and 0.2 were applied.

## 3 RESULTS

### 3.1 Biobank‐based overlap simulation results indicates PRS bias

#### 3.1.1 UKBB CAD simulation

We first conducted a series of simulation studies using UKBB CAD data to investigate the bias in PRS due to sample overlap, with CAD as the phenotype (Figure S2). The results indicated a substantial risk of overfitting even when the overlap is small. In these simulations, the UKBB CAD data were split into training and target datasets with varying ratios (50:50, 60:40, 70:30, 80:20, and 90:10), and various increments of samples from the target dataset (1%, 3%, 5%, 10%, 20%, 40%, 60%, and 90%) were added to the training dataset to create overlap samples. The AUROC for the PRS without sample overlap was 0.785 when 50% of the data was used for training and the other 50% as the target dataset. As sample overlap increased, the AUROC also increased, reaching a maximum value of 0.93 (Figure 2A). This pattern was consistent across different training–target dataset ratios and PRS construction methods (Figure S4 and Tables S1–S4 in supporting information).

Following the meta‐inverse approach, we applied IVW‐based and *Z* score–based methods to the summary statistics generated from the training and overlapped datasets to address this issue. We found the two meta‐inverse approaches in OA PRS to effectively control sample overlap bias across all the different data splits and overlaps tested; when using a 50:50 split ratio, the mean AUROC was 0.579 (range: 0.570–0.583), as shown in Figure 2A and Table S2. The *Z* score–based method controlled bias best in most cases, and the IVW‐based method followed closely behind (Figures 2A and S5 in supporting information). We also assessed the EraSOR method, which is presently the only method available for adjusting sample overlap without knowledge of the overlapped individuals.^29^ When looking at AUROC on the whole target dataset, EraSOR slightly overadjusted the PRS as the overlap proportion increased (Figure 2A). However, when we separately investigated AUROC values on the overlap and non‐overlap samples from the target dataset, EraSOR showed poor performance. Specifically, EraSOR tended to produce inflated AUROCs on overlap samples and overadjusted AUROCs on non‐overlap samples (Figure 2A). EraSOR also showed slightly lower ORs per SD in all scenarios; this was especially evident with a large target sample (50%) and high overlap (90%; Figure S3, Table S5 in supporting information).

To evaluate the performance of the methods when the sample size of the target data is small, which is very common in many studies using PRS, we randomly sampled from UKBB CAD data to build a training set consisting of 30,000 controls and 8000 cases and a target set with 500 controls and 500 cases. We selected overlapping samples from the training set and added them to the test set in increments of 100, 200, 300, 400, and 500 cases and controls. We performed this simulation 10 times, randomly sampling each time, and evaluated AUROC. As the overlap between the training and target datasets increased, we observed an inflation in AUROC in the target dataset. However, the meta‐inverse approaches could still adjust for overfitting, whereas EraSOR exhibited inflated AUROC on overlap samples and overadjusted AUROCs on non‐overlap samples (Figure 2B, Table S6 in supporting information). This result suggests that the OA PRS method is robust to a low sample size in the target dataset.

#### 3.1.2 AGEN‐T2D and KoGES analysis

The AGEN‐T2D consortium consists of 433,540 samples from 23 East Asian genetic studies, including 93,691 samples from the KoGES. KoGES samples were adjusted and tested for inflation on PRS. Manhattan plots on IVW‐adjusted effects (Figure S6 in supporting information) show that some outlier markers appeared that were not present on both GWAS. This phenomenon is likely due to the unstable variance estimation in the IVW‐based method, in which the consortium and target GWAS have similar SDs.

**FIGURE 3:**
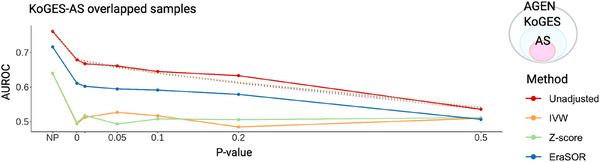
Visual diagnostics for the adjustment using AGEN‐T2D—KoGES samples. Only the AS cohort (*n* = 5490) in KoGES was adjusted. In this case, there are no results on non‐overlapped samples because the KoGES study completely includes the AGEN‐T2D consortium. AGEN‐T2D, Asian Genetic Epidemiology Network Type 2 Diabetes; AS, Ansan/Ansung; AUROC, area under the receiver operating characteristic; EraSOR, Erase Sample Overlap and Relatedness; IVW, inverse variance weighting; KoGES, Korean Genome and Epidemiology Study.

PRS created by overlap adjustment methods showed that the EraSOR has higher AUROC results than the meta‐inverse approaches. To investigate such differences, we applied visual diagnostics after adjusting a part of the KoGES samples, the AS cohort. The AUROC of adjusted PRS suggests that the adjustment did not impact other overlapped KoGES samples but reduced only the overfitting of AS samples (Table S7 in supporting information). Specifically, the diagnostic plot showed the IVW and *Z* score methods to achieve an AUROC of 0.5 at *P* = 0.05; however, values for EraSOR and the original PRS CS score remained > 0.6 and 0.7, respectively (Figure 3).

### 3.2 OA PRS adjust overlap bias between ADNI and IGAP

We evaluated overfitting using the IGAP consortium data, which has 63,926 samples. Among those, 327 samples originated from the phase 3 ADNI dataset (which totals 1040 AD samples). Existing AD PRS studies have used IGAP as training data and ADNI as target data.^2^, ^3^, ^30^ We trained a PRS using IGAP summary statistics and obtained an AUROC of 0.915 (OR per SD = 20.21) for the 327 overlapped samples. However, when the PRS model was applied to the remaining 713 ADNI samples not included in IGAP, the AUROC decreased to 0.726 (OR = 4.34). This severe inflation of area under the curve among overlapped samples indicates the need to adjust for the inclusion of ADNI samples in the IGAP consortium.

**FIGURE 4:**
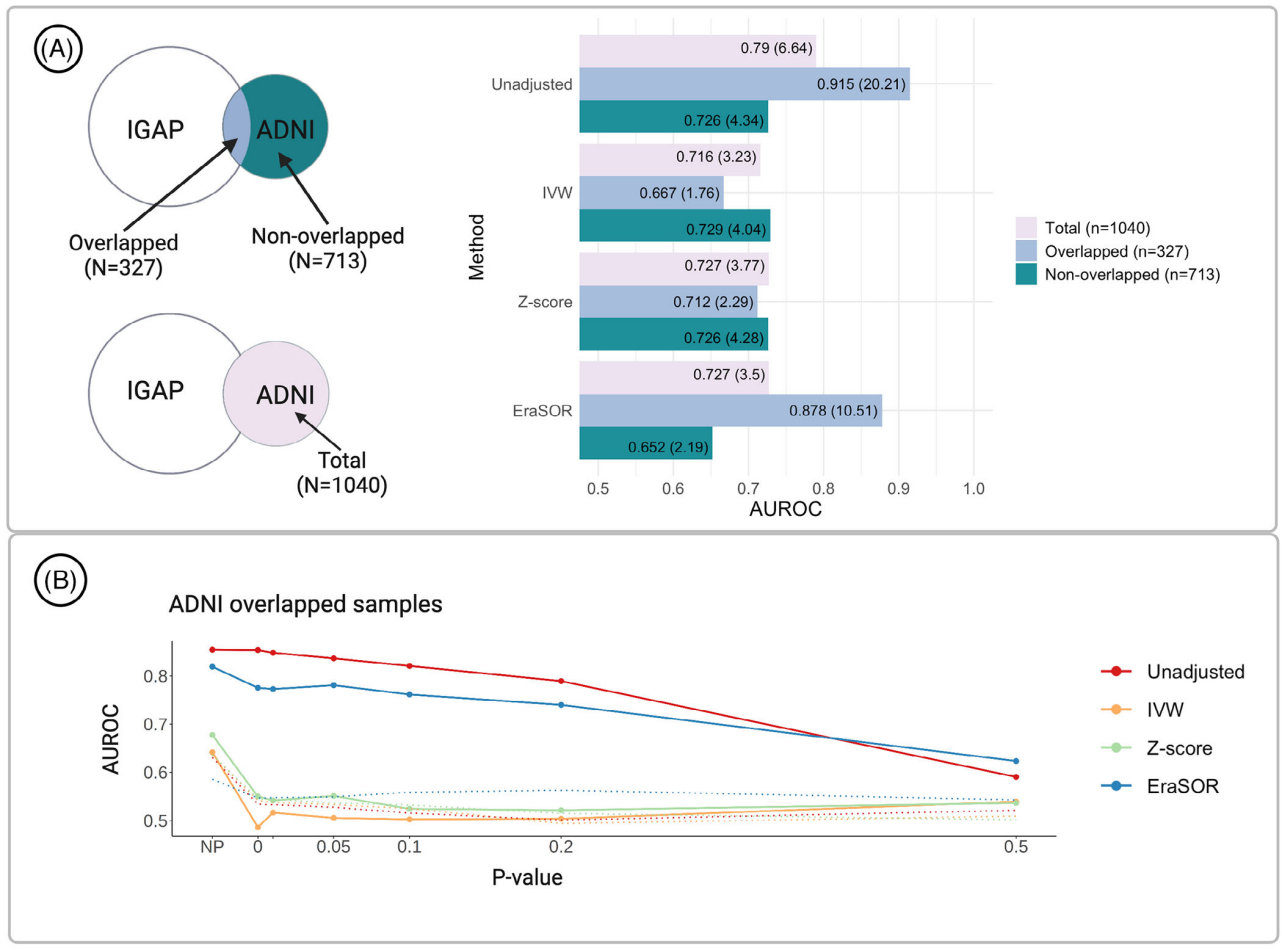
Results from real data analysis using the IGAP—ADNI. A, Inflation of AUROC values in overlap, non‐overlap, and target (overlap + non‐overlap) IGAP—ADNI samples without and with adjustment by three methods—IVW, *Z* score, and EraSOR. The corresponding OR values are in the parentheses. B, Visual diagnostics for the analysis in (A). High significant variants and neighbors are filtered at *P* = 1e‐4. The dotted trajectory represents AUROC from non‐overlapped samples. ADNI, Alzheimer's Disease Neuroimaging Initiative; AUROC, area under the receiver operating characteristic; EraSOR, Erase Sample Overlap and Relatedness; IGAP, International Genomics of Alzheimer's Project; IVW, inverse variance weighting; OR, odds ratio.

Next, we applied OA PRS to IGAP and ADNI data. This yielded AUROC values of 0.667 (OR = 1.76) with IVW and 0.712 (OR = 2.29) with the *Z* score method on overlapped samples, which were close to those obtained for non‐overlapped samples (Figure 4A, Table S8 in supporting information). A diagnostic visualization of sample overlap adjustments from OA PRS is presented in Figure 4B.[Fig alz70109-fig-0005] As the *P* value threshold was increased to 0.2, AUROC values for both IVW and *Z* score methods approached 0.5.

#### 3.2.1 Drawbacks of removing overlap samples from the ADNI cohort

One common practice to solving the sample overlap problem is to remove the overlapping samples from the target dataset. However, this strategy is not viable if the entire dataset is encompassed within the training dataset as in the case of AGEN‐T2D and KoGES. Additionally, it is important to note that we can gain more power by incorporating overlapped samples without overfitting risk rather than removing them.

**FIGURE 5 alz70109-fig-0005:**
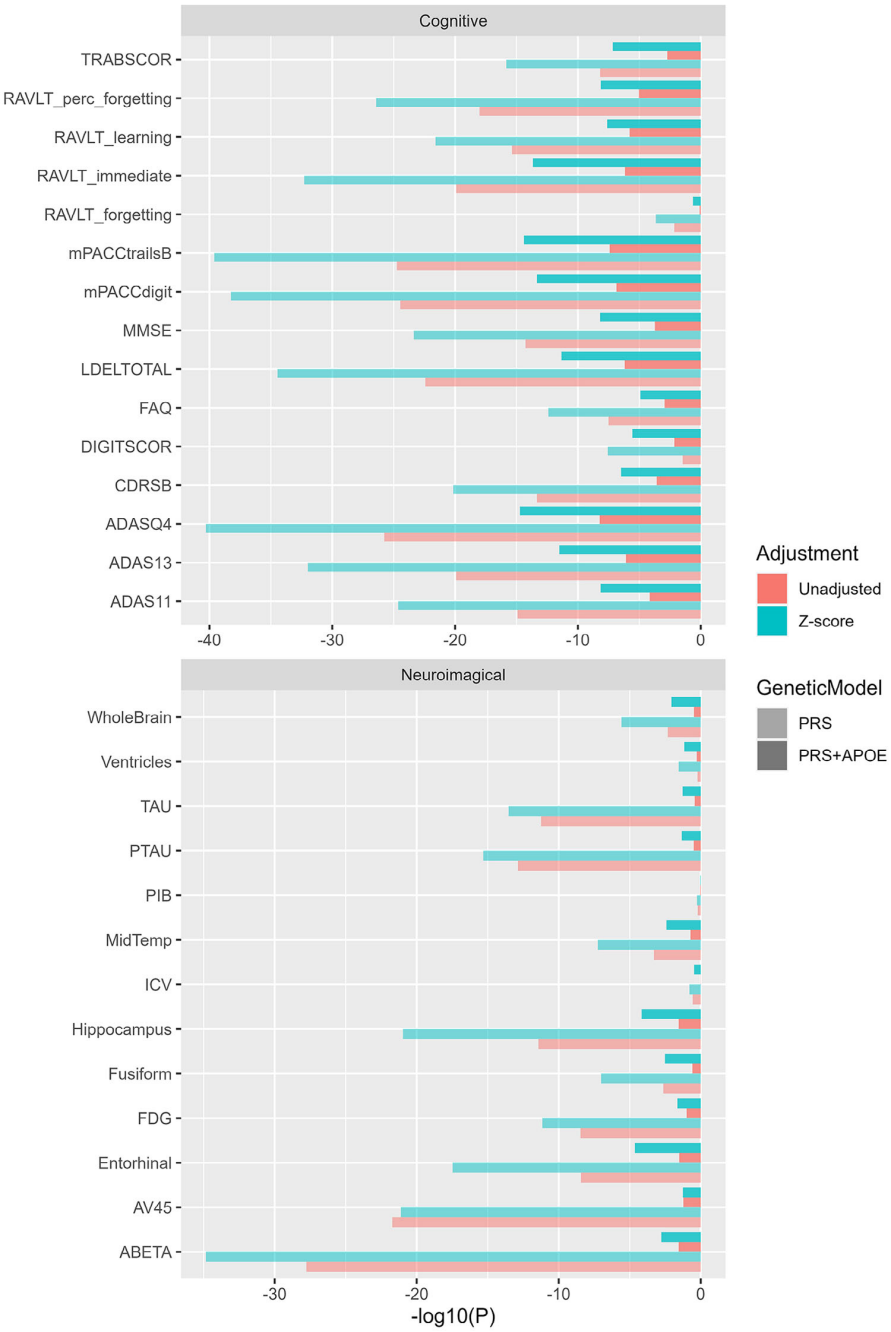
Model –log10 *P* value of AD PRS for predicting 28 cognitive and neuroimaging phenotypes. Overlapped samples were removed from unadjusted PRS while *Z* score adjustment included all samples. On both models which incorporate either PRS only or PRS and *APOE* genotypes, *P* values were lower when using adjusted AD PRS. AD, Alzheimer's disease; *APOE*, apolipoprotein E; PRS, polygenic risk score.

The association level of PRS with 28 brain‐related phenotypes, representing clinical, magnetic resonance imaging, positron emission tomography, and biospecimen data from ADNI, was evaluated. The statistical significance of the AD PRS score increased in all categories with the *Z* score, contrasted with the exclusion of overlapping samples and the use of the unadjusted AD PRS (Table S9 in supporting information). The –log10 *P* value plot shows that additional samples from the overlapped set contribute to building a stronger association between PRS and related phenotypes (Figure 5). The increment remained as the apolipoprotein E ε4 genotype variable is included in the model, and adjusted *R* squared also showed a higher explanation of PRS to phenotypes (Figure S7 in supporting information). Standard errors of AD PRS coefficients on most phenotypes were reduced, indicating the power increase of having 350 overlapped samples in addition to the 1195 non‐overlapped samples (Figure S8 in supporting information).

## 4 DISCUSSION

This study aims to perform an extensive investigation of overfitting bias due to sample overlap with present consortia and their participating GWAS. The experimental results from the UKBB data showed that the overfitting bias due to sample overlap could be substantial even if the target data size is small, indicating the necessity of overlap adjustment. With the application of OA PRS, we were able to effectively adjust overlapping ADNI samples from IGAP summary‐based PRS, enabling them to be used for extended research without overfitting. The researchers can either use the adjusted summary statistics generated in this study or could apply OA PRS themselves to adjust for sample overlaps.

As part of OA PRS, we suggested a meta‐inverse approach, which excludes the overlapped samples effect from the GWAS summary data by inverting the meta‐analysis procedure and using the overlap‐adjusted GWAS summary data to construct PRS. By comparing to the de‐correlation approach, we showed that the meta‐inverse could robustly adjust for the overlap bias regardless of the sample size. Further, the *Z* score–based method was robust even when adjusted variance estimation failed. In contrast to the previous approach, EraSOR, OA PRS consistently controlled AUROC across the entire target dataset, eliminating bias in overlap and non‐overlap samples. After the overlap adjustment, evaluating whether the modification resolves the overfitting is essential. For such purposes, visual diagnostics can be used to compare PRS with and without adjustment. With OA PRS, one can robustly construct PRS with any existing PRS construction methods.

OA‐PRS also has limitations. Because the *Z* score–based method relies on MAF for estimating standard error and effect sizes, using data‐specific MAF is recommended when precise effect estimates are needed. Also, the accurate identification of overlapped samples is pivotal in OA PRS, a task that often presents considerable difficulty in consortium studies. In response, we plan to develop methods to address these problems in future studies. Additionally, as the future roadmaps of the ADNI and IGAP studies evolve, ongoing tasks include updates and adjustments based on newly identified overlapped samples. Ultimately, by investigating the bias due to sample overlap and providing a procedure to address it, this work will help construct reliable PRS for integrative downstream analysis.

## 5 CONCLUSION

Our study revealed that overfitting bias due to sample overlap could be substantial, even with minimal sample overlap or in small target datasets. To address this, we introduced OA PRS, a sample overlap adjustment procedure with visual diagnostics. The traditional adjustment approach was subject to bias in the case of overlap between the ADNI and IGAP datasets, whereas the suggested OA PRS was able to resolve overfitting due to overlap. We also demonstrate that a simple diagnostic procedure in OA PRS can help verify whether the adjustment was made correctly. By applying OA PRS to adjust the IGAP summary statistics, it became feasible to use extensive features of ADNI along with PRS, eliminating the risk of overfitting.

## AUTHOR CONTRIBUTIONS

Seokho Jeong and Manu Shivakumar contributed equally as first authors; they analyzed the data and wrote the manuscript. Dokyoon Kim and Seunggeun Lee supervised the data analyses and revised the manuscript. Sang‐Hyuk Jung provided assistance in extraction of UKBB data. Hong‐Hee Won, Kwangsik Nho, Heng Huang, Christos Davatzikos, Andrew J. Saykin, Paul M. Thompson, and Li Shen provided technical expertise in curation of ADNI data. Young Jin Kim and Bong‐Jo Kim contributed to analyzing KoGES data. All authors critically reviewed the manuscript and contributed important intellectual content. All authors have read and approved the final manuscript as submitted.

## CONFLICT OF INTEREST STATEMENT

The authors have nothing to disclose. Author disclosures are available in the supporting information.

## CONSENT STATEMENT

All participants provided informed consent in their respective cohorts used in this study. The ethics approval was obtained from the relevant authorities. This study was performed using data from the UK Biobank, KoGES, and ADNI. The study was performed in accordance with the Declaration of Helsinki.

## Supporting information



Supporting Information

Supporting Information

Supporting Information

## Data Availability

The IGAP summary statistics after adjusting overlapped ADNI samples with OA‐PRS are available through the OA‐PRS GitHub repository—https://github.com/leelabsg/OAPRS. IGAP summary statistic is available at—https://www.niagads.org/datasets/ng00075. *OAPRS* R package via Github—https://github.com/leelabsg/OAPRS.
