# Supplementary material for: Addressing overfitting bias due to sample overlap in polygenic risk scoring
Source: Alzheimers Dement. 2025 Apr 6;21(4):e70109. doi: 10.1002/alz.70109 (PMC11972974; doi:10.1002/alz.70109)

**Supplemental Information**

Supplemental Methods

​​PRS generation methods

PRS was generated using PRS-CS, PRSice2, and LDPred2. The UKBB CAD simulations and IGAP and ADNI overlap analysis were carried out using all three methods. For PRS-CS, we used the European LD reference panel constructed using the 1000 Genomes Project phase 3 samples provided on the PRS-CS GitHub page (<https://github.com/getian107/PRScs>). For LDPred2, we used the HapMap3+ variants with independent LD blocks, which the authors recommended. PRSice2 was run with the default parameters.

Visual Diagnostics generation methods

The first step of visual diagnostics is removing highly associated variants (p<1e-4) and their linkage disequilibrium blocks. We eliminated all variants from such LD using Estimated independent LD blocks. The independent LD lists are stored in BED and are available in both hg37(<https://bitbucket.org/nygcresearch/ldetect/src/master/>) and hg38(<https://github.com/jmacdon/LDblocks_GRCh38>). In the second step, we generated the filtered list on multiple p-value thresholds.

Simulations

**Settings**

With the assumption of 10% minor allele frequency, genotypes were generated from Bin(2,0.1). Normal and binomial distributions were used to generate continuous and binary phenotypes, respectively. Both phenotypes were generated under the null assumption.

**Results**

**
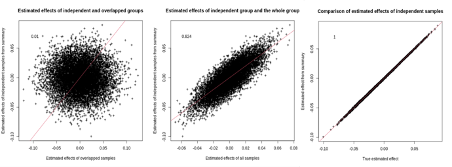
**

After applying adjustment on simulated genotypes, the adjusted and actual estimated effects had high concordance (Figure (c)). Also, overlapped results showed no correlation with the adjusted effects, while concordance between all sample effects and adjusted effects (Figure (a),(b)).

To discover how OA-PRS performs in cases where data-specific MAF and reference panel based MAF, we gave different allele frequencies in overlapped and non-overlapped samples to the simulation setting. Although OA-PRS does not rely on MAF information when estimating standard effect size, standard error may vary when MAF is misspecified or different across cohorts. The scenarios included relative differences of ranging from 0.5 to 1.5 on 1%, 5%, 10%, 20%, and 30% of non-overlapped MAF. shows correlation (R2) between actual standard effect size and estimated effects from IVW and Z-score based methods in OA-PRS ( Supplementary Figure S1). The result shows estimation using OA-PRS does not vary from actual effect size when MAF is different within rare (1%) to common variants.

Variant and Sample QC methods

**UKB**

The available UKB samples with imputed genotype data are 487,409. We removed poor-quality samples that the UKB provided. We dropped all second-degree related individuals by choosing the minimum number of individuals from the related pairs provided by UKB using a greedy algorithm. Samples that mismatch between reported sex with genetically inferred sex was eliminated. We filtered all variants with an INFO score < 0.3 and MAF < 0.01. By extracting only the European population, the final data used in this analysis contained 377,921 samples and 9,505,767 variants.

**ADNI**

WGS genotyping was performed using HiSeq2000 using a read length of 100bp and then the genome assembly with GRCh38 as the reference genome. Primary quality control steps using the SNP/Indel Variant Calling Pipeline (VCPA), which was developed and maintained by the National Institute on Aging Genetics of Alzheimer's Disease Data Storage Site (NIAGADS), including SNV concordance check, sex mismatch, contamination check, and relatedness (Pihat > 0.4) were performed. During additional quality control, multi-allelic variants, monomorphic variants, and variants with GQ < 20 and DP < 10 were removed. The QC was performed by the Genome Center for Alzheimer’s Disease (GCAD) Data Production Team. We further filtered out samples with a missing rate > 95%, variants with a missing rate > 90%, and variants that violate Hardy-Weinberg equilibrium (p < 1.0 × 10−6). Among 15,456,635 variants and 1545 samples passed the QC, we used 1040 samples with AD phenotype or no related disease. After removing samples due to IBD (n=3), 327 overlapped with the IGAP dataset. 28 variables on the baseline from the ADNI cohort were also used in this analysis which were obtained from Image and Data Archive (IDA) run by Laboratory of Neuro Imaging (LONI)(32, 33).

**Agen and KoGES**

Agen summary statistics on T2D are gathered from up to 433,540 participants with 11,825,585 markers from 23 GWAS, including z-score, effect size, standard error, minor allele frequency, and p-value. BMI-unadjusted GWAS effects were used (). In each study, variants with mismatched chromosomal positions or alleles, ambiguous alleles (AT/CG) with high minor allele frequency (> 40%), or significant allele frequency differences (>20%) compared to East Asian-specific allele frequencies were filtered out. The imputation was performed using minimac3 or IMPUTE2 with 1000 Genomes Project data as reference panels.

Genotyped using the KoreanChip array, KoGES samples were excluded under the following criteria: low call rate (< 97%), excessive heterozygosity, excessive singletons, gender discrepancy, and cryptic first-degree relatives. SNPs with low HWE p-value (< 10−6) or low call rate (< 95%) were excluded. Then, called genotypes were phased using Eagle v2.3 and imputed using IMPUTE4 with 1000 Genomes Project Phase 3 data and the Korean reference genome as a reference panel. Imputed variants with low imputation quality scores (< 0.8) and MAF (< 1%) were excluded.

Supplemental Figures

**Supplementary Figure S1:** Correlation(R^2^) between actual effect size and estimate from

OA-PRS. a) The x-axis represents relative minor allele frequency of overlapped samples

compared to non-overlapped samples. b) The x-axis represents minor allele frequency ranging

from 1% to 30%.


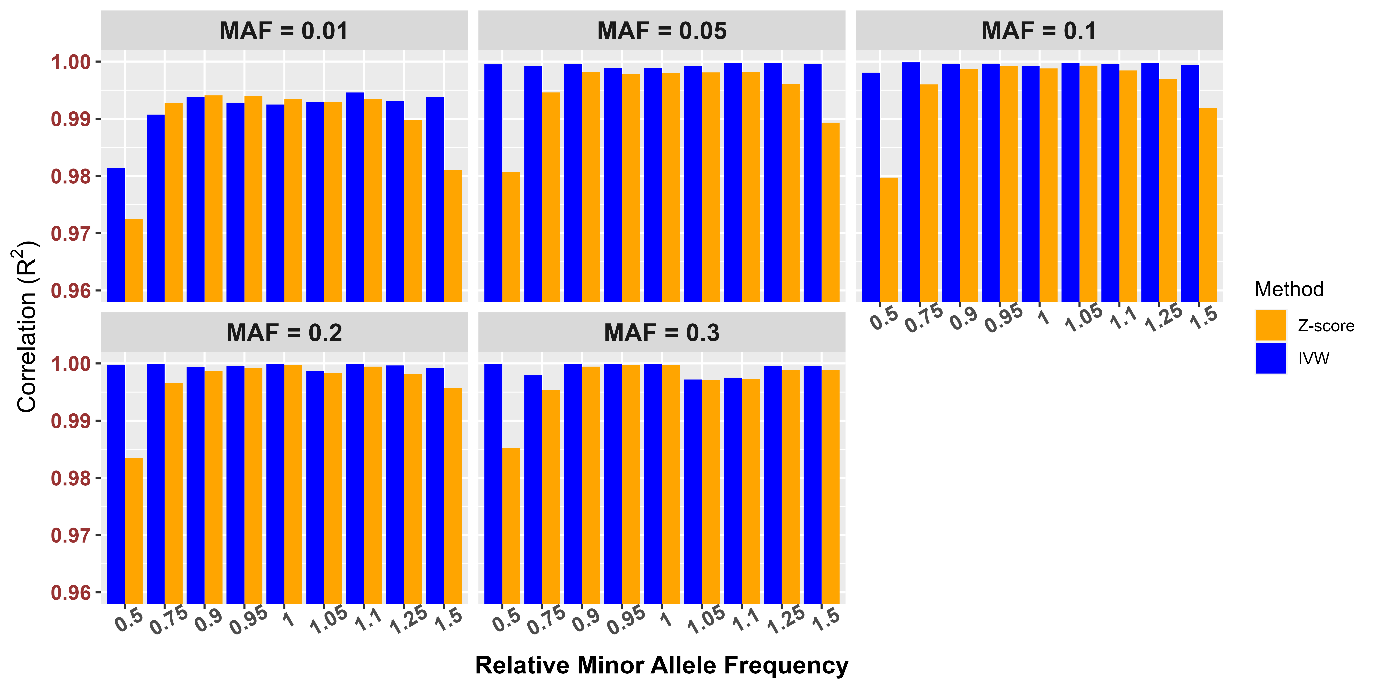


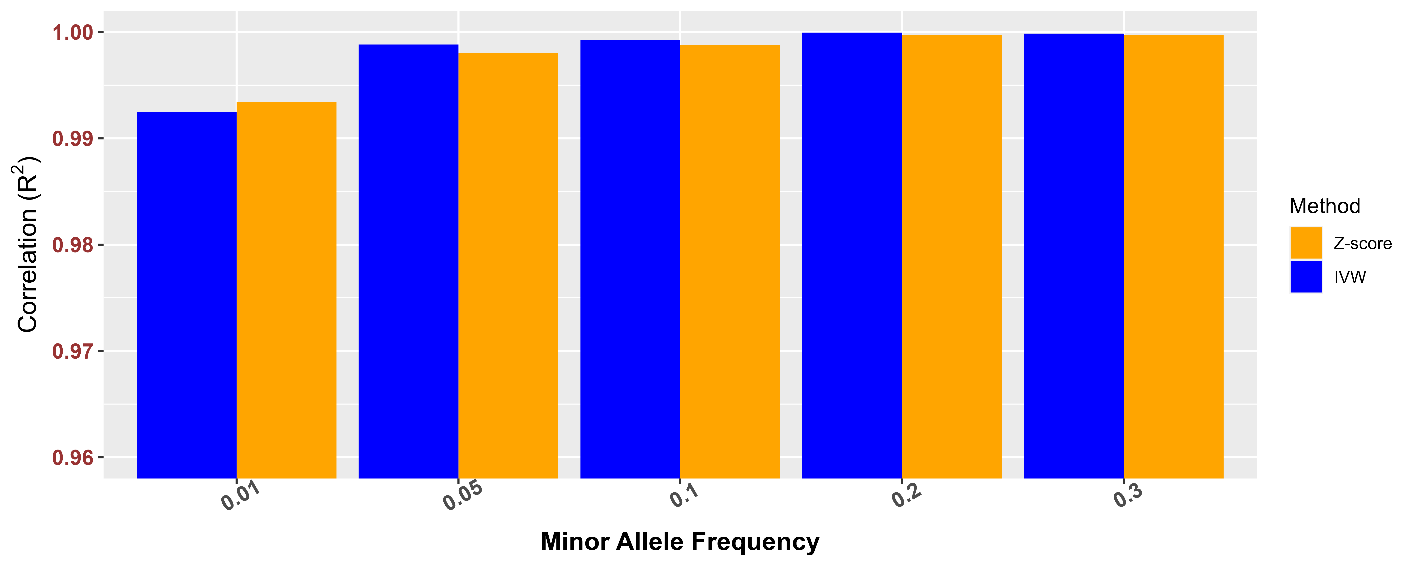


**Supplementary Figure S2**: Workflow of the UKB CAD simulations

**
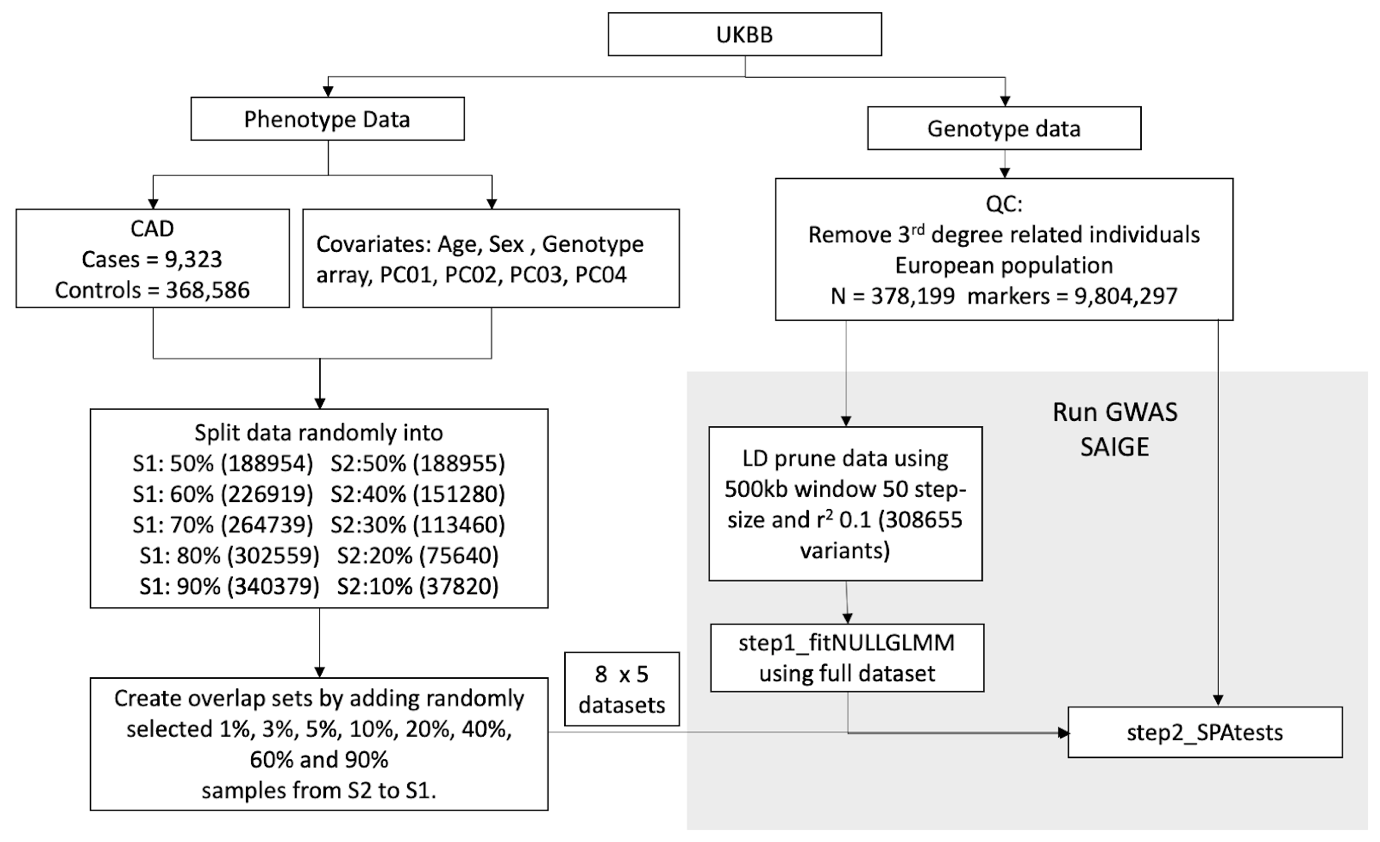
**

**Supplementary Figure S3:** Results from simulation studies using the UKB-CAD dataset showing inflation in OR per standard deviations (SD) values for the target (overlap + non-overlap) data as the proportion of overlap samples increases and with adjustment by three different methods - IVW, Z-score, and EraSOR. Training and target data splits are shown above the plots (50:50, 60:40, 70:30, 80:20, and 90:10). The Y-axis plots the OR per SD values and the X-axis the percentage of overlap samples from the target dataset (1%, 3%, 5%, 10%, 20%, 40%, 60%, and 90%).

**
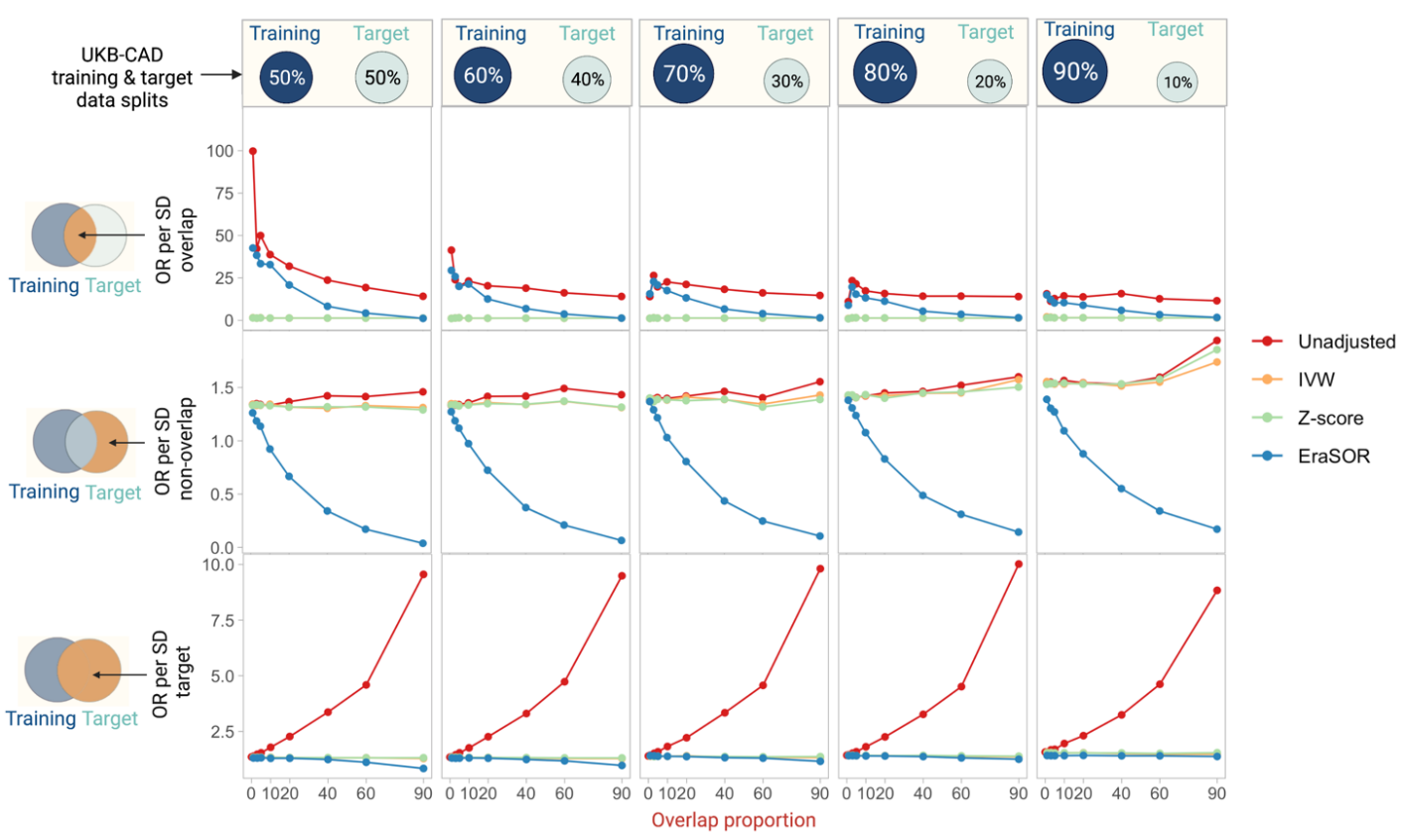
**

**Supplementary Figure S4:** AUC inflation with different overlap proportions using LDPred-2, PRS-CS, and PRSice-2.


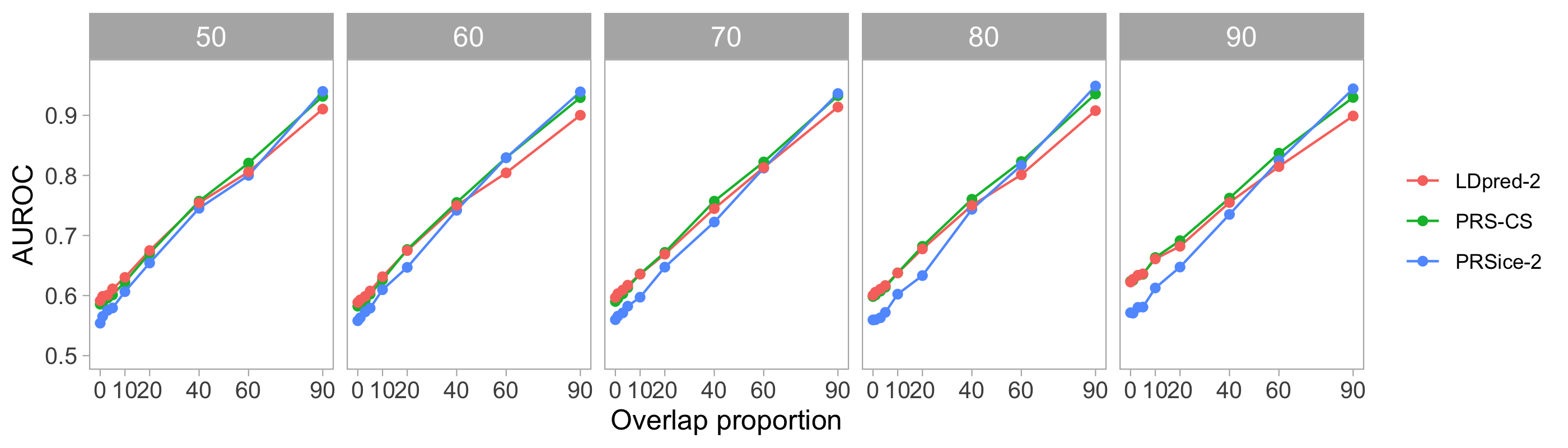


**Supplementary Figure S5:** Inflation in AUROC as a proportion of overlap samples increases in CAD UKBB dataset and inflation after adjustment using OA_PRS using PRsice2 and Ldpred2 for generating PRS scores.


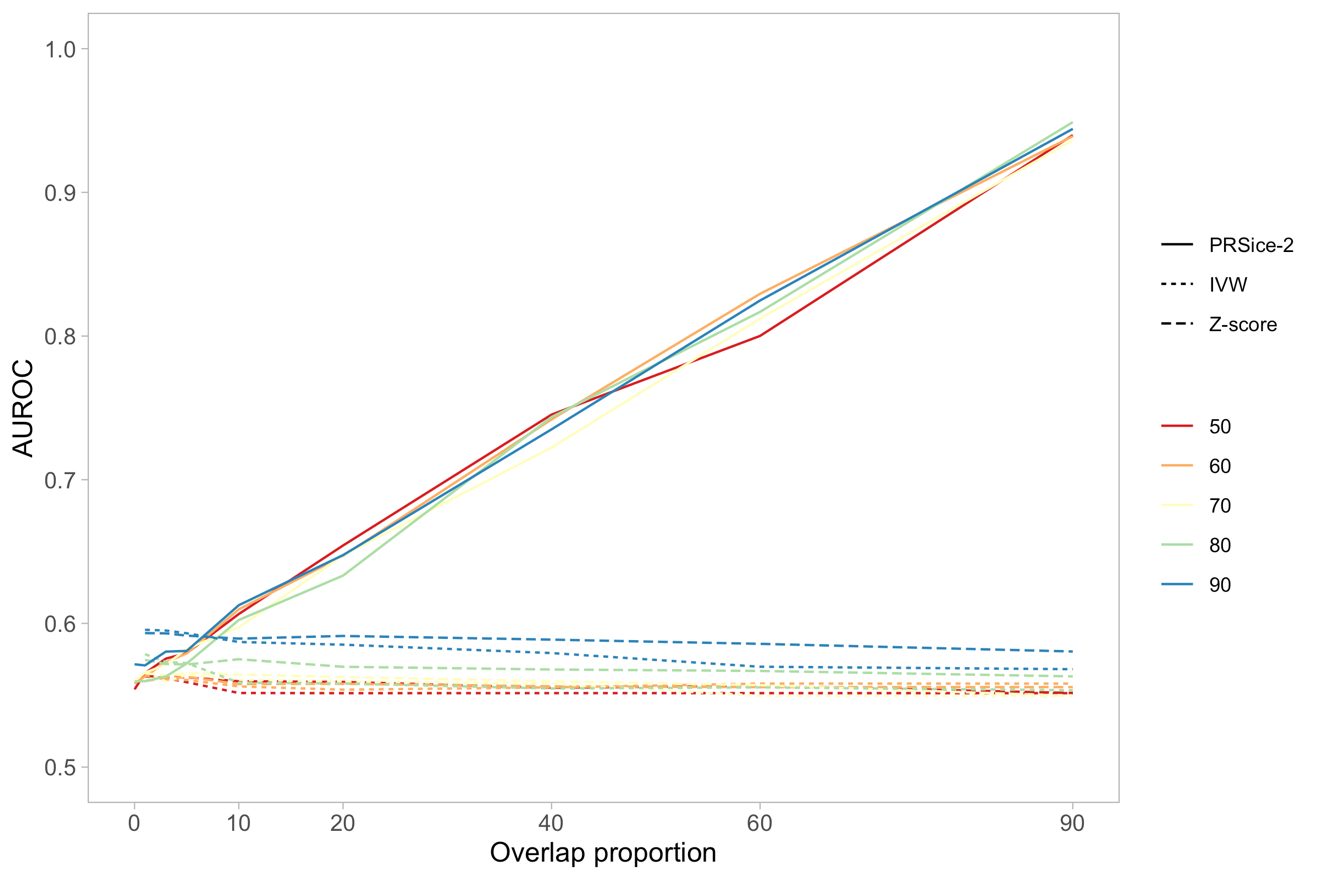


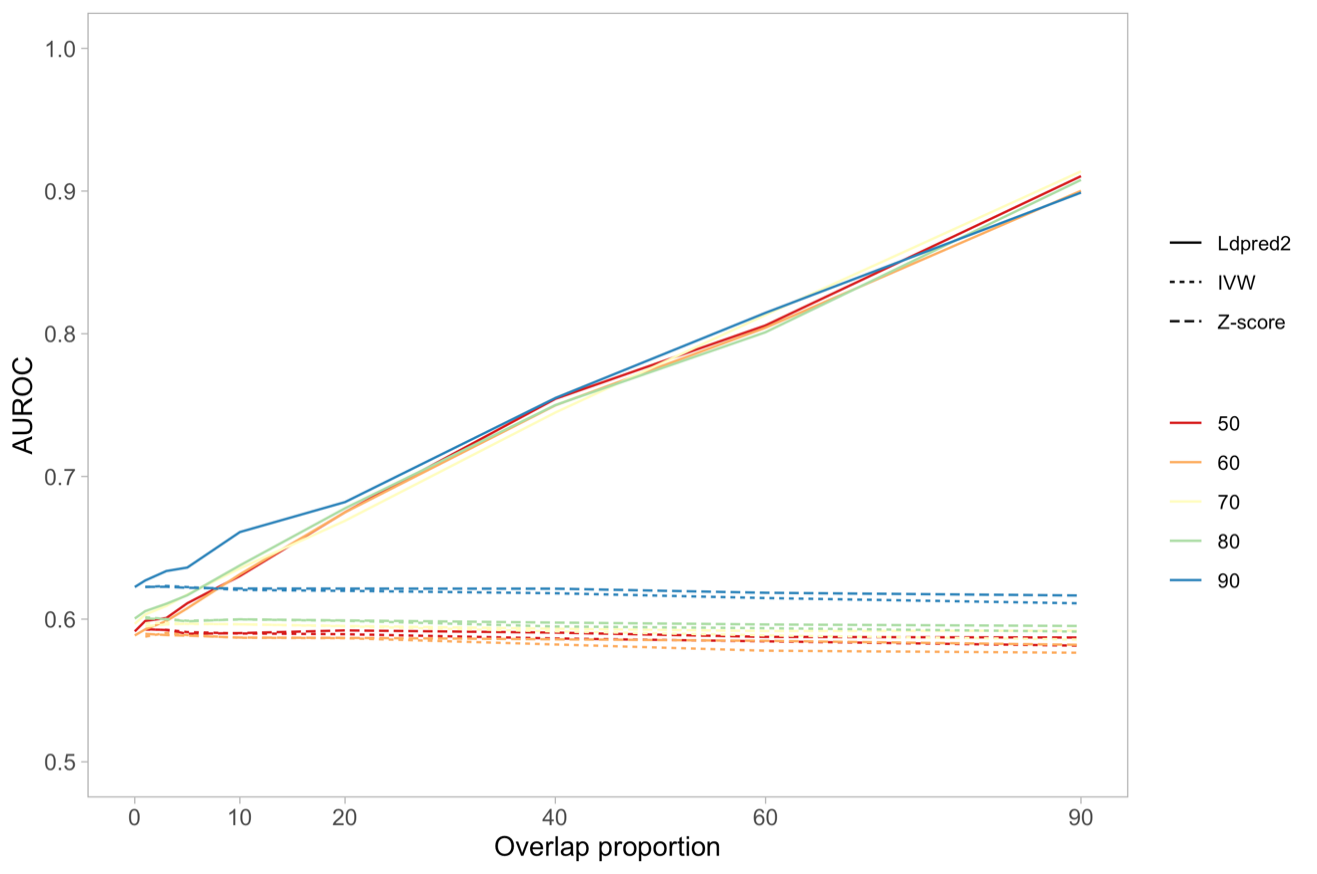


**Supplementary Figure S6:** Manhattan plots of adjusted summary statistics of Agen-T2D. The first plot indicates randomly associated loci from the IVW-based method—the second plot results from the Z-score-based method, showing no visible outliers or peaks.


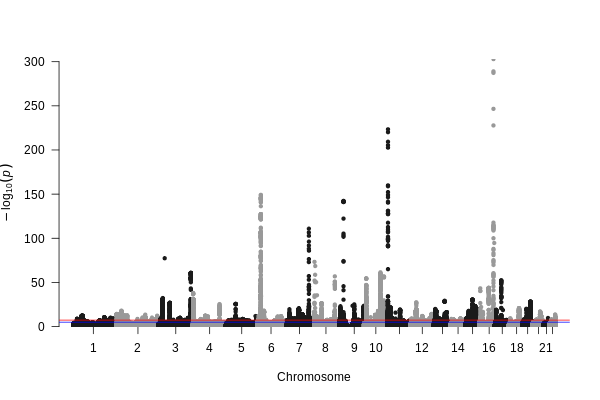

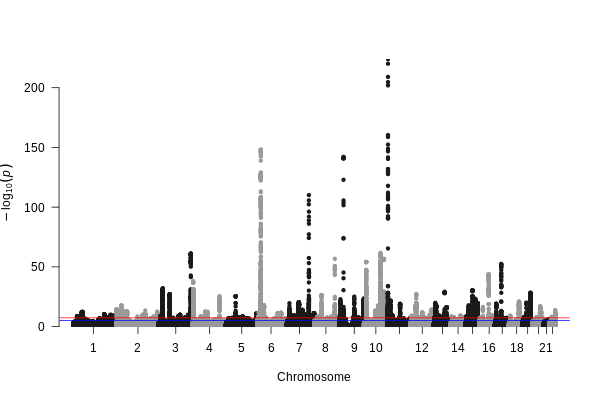


**Supplementary Figure S7**: Effects (Adjusted R**^2^**) of AD PRS regression model for predicting 28 phenotypes. AD PRS with APOE genotype model also indicates adjusted AD PRS leads to higher explanation of model to extensive phenotypes in ADNI.

**
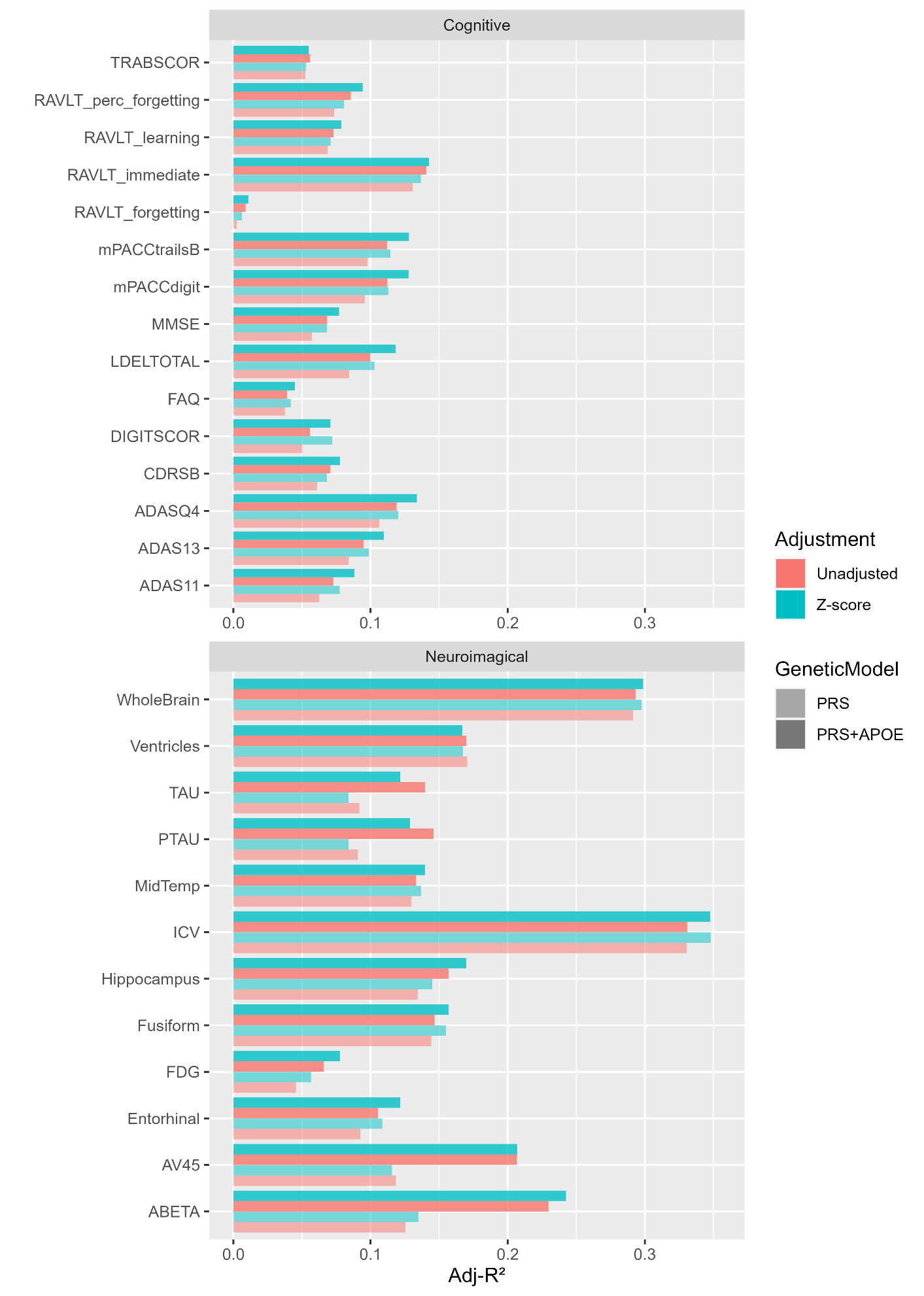
**

**Supplementary Figure S8**: Relative Standard error of effect size on AD PRS for each phenotype.


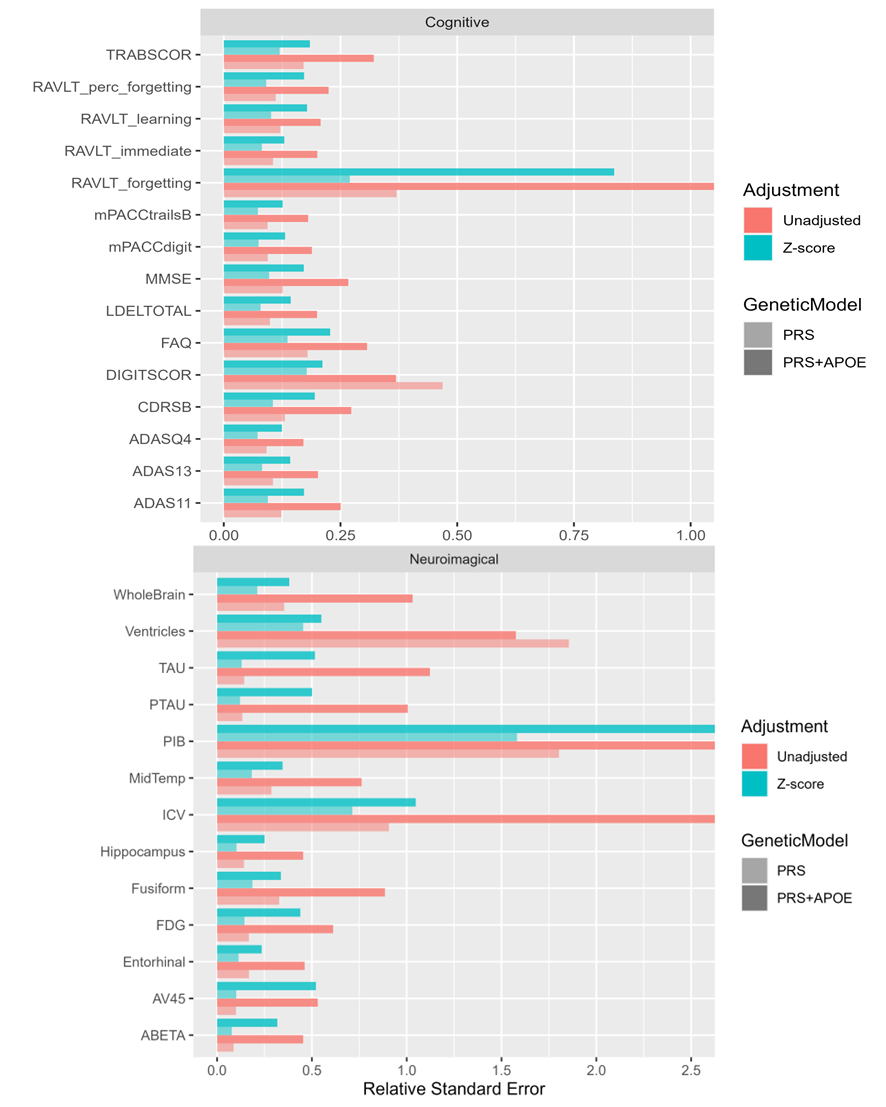

Supplement: Supplementary file 1 — Supporting Information [file ALZ-21-e70109-s002.docx]
